# Ablação de Curta Duração de Alta Potência ou Ablação de Campo Pulsado: Qual é a Melhor Escolha para Ablação da Fibrilação Atrial?

**DOI:** 10.36660/abc.20250057

**Published:** 2025-03-25

**Authors:** José Carlos Pachon-M, Enrique I. Pachon-M, Tasso J. Lobo, Tomas G. Santillana-P, Carlos Thiene C. Pachon

**Affiliations:** 1 Faculdade de Medicina da Universidade de São Paulo São Paulo SP Brasil Faculdade de Medicina da Universidade de São Paulo, São Paulo, SP – Brasil; 2 Hospital do Coração São Paulo SP Brasil Hospital do Coração, São Paulo, SP – Brasil

**Keywords:** Ablação por Cateter, Arritmias Cardíacas, Fibrilação Atrial, Terapia de Eletroporação Irreversível

Elogiamos os autores por sua valiosa contribuição ao estudo "Ablação de Fibrilação Atrial: Eletroporação versus Ablação por Radiofrequência de Alta Potência e Curta Duração",^1^ que investiga a eficácia e a segurança de duas técnicas distintas de ablação — radiofrequência de curta duração e alta potência (HPSD)^2^ e ablação por campo pulsado (PFA)^3^ — para o tratamento de fibrilação atrial (FA) sintomática. Realizado em um único centro em 2022, o estudo incluiu 101 pacientes, 75% homens, com idade média de 61 anos. Este estudo é particularmente oportuno e relevante, pois tanto HPSD quanto PFA são técnicas de ablação de FA relativamente novas que necessitam de avaliação abrangente para estabelecer melhor sua utilidade clínica, indicações e perfis de risco em comparação com tecnologias convencionais bem estabelecidas.

Os resultados indicaram que ambas as técnicas efetivamente alcançaram o isolamento das veias pulmonares (IVP), com HPSD mostrando tempo de fluoroscopia significativamente menor em comparação com PFA (5 vs. 13 minutos). No entanto, HPSD teve uma duração de procedimento mais longa (97 vs. 88 minutos). As complicações foram mínimas, com apenas uma complicação principal (tamponamento cardíaco) ocorrendo no grupo PFA.

Durante o acompanhamento com média de 384 dias, 75% dos pacientes mantiveram o ritmo sinusal, com recorrência de FA observada em 25% dos casos. Embora a PFA tenha demonstrado tempos de procedimento mais curtos e taxas de recorrência potencialmente menores, ambos os métodos provaram ser seguros e eficazes para o tratamento da FA. Este estudo destaca a avaliação contínua de novas tecnologias de ablação na prática clínica.

Embora A HPSD seja uma modificação da conhecida ablação por radiofrequência (RF),^4^ PFA é uma técnica não térmica emergente para ablação de FA que utiliza pulsos elétricos de alta voltagem (até 2000 V) para induzir eletroporação irreversível, visando seletivamente o tecido cardíaco e minimizando os danos às estruturas circundantes.^5 , 6^ Estudos recentes se concentraram principalmente na segurança e eficácia do PFA, com atenção especial aos eventos tromboembólicos.

No estudo pivotal PULSED FA,^6^ a PFA demonstrou uma baixa taxa de eventos adversos de segurança primária (0,7%), sem casos relatados de estenose de VP, lesão do nervo frênico ou lesão esofágica. O estudo concluiu que o PFA fornece eficácia consistente com tecnologias de ablação estabelecidas, ao mesmo tempo em que oferece um perfil de segurança favorável.

Entretanto, desenvolvimentos recentes levantaram preocupações quanto aos riscos tromboembólicos associados ao PFA.^3 , 7^ Johnson & Johnson suspendeu temporariamente as vendas do seu sistema PFA, Varipulse,^8 - 10^ depois que quatro pacientes apresentaram eventos neurovasculares, identificados como derrames, após o tratamento. Este incidente levou a um maior escrutínio dos sistemas PFA e destacou a necessidade de uma avaliação completa de seus perfis de segurança.

Esses eventos ressaltam a importância da pesquisa contínua e da vigilância pós-comercialização para entender completamente os riscos tromboembólicos associados ao PFA. Embora estudos iniciais indiquem um perfil de segurança favorável, eventos adversos recentes sugerem que mais investigação é necessária para garantir a segurança do paciente.

Em resumo, embora o PFA ofereça vantagens promissoras na ablação de FA, incluindo redução de danos colaterais e formação eficiente de lesões, relatos recentes de eventos tromboembólicos destacam a necessidade de vigilância e pesquisa contínuas para avaliar e mitigar totalmente os riscos associados.

Como o estudo atual comparou HPSD vs. PFA,^1^ é altamente apropriado avaliar cada uma dessas tecnologias individualmente em relação à ablação convencional por cateter de RF. A Tabela 1 fornece uma visão geral das metanálises mais recentes que avaliam essas tecnologias.

**Tabela 1:** Comparação entre metanálises de ablação de FA utilizando HPSD e PFA

Parâmetro	Metanálise HPSD (Xu et al., 2022)^11^	Metanálise de PFA (Aldaas et al., 2024)^12^
**Objetivo**	Comparar a ablação HPSD (>40 W) vs. ablação LPLD para FA, com foco na eficácia, segurança e eficiência do procedimento.	Comparar PFA com ablação térmica (RF/crio) para FA, com foco na eficiência, segurança e eficácia do procedimento.
**Número de Estudos**	15 estudos	6 estudos
**Total de pacientes**	3.255 pacientes (1.780 HPSD, 1.475 ablação térmica/RF/crio)	1.012 pacientes (441 PFA, 571 ablação térmica/RF/crio)
**Resultados primários**	Recorrência de ATAs, recorrência de FA, IVPP, RVP aguda, tempo de procedimento, tempo de fluoroscopia, ETI, complicações.	Tempo de procedimento, tempo de fluoroscopia, complicações periprocedimentais, recorrência de FA.
**Recorrência de FA/ATAs**	Menor recorrência de ATAs no acompanhamento de 1 ano (OR: 0,49; IC 95%: 0,35–0,67, p<0,0001).	Nenhuma diferença significativa na recorrência de FA (RR: 0,64; IC 95%: 0,31–1,34).
**Tempo Procedimental**	Menor tempo de procedimento (DMP: -0,95; IC 95%: -1,06 a -0,85, p<0,00001).	Menor tempo de procedimento (DM: -21,95 minutos; IC 95%: -33,77 a -10,14, p=0,0003).
**Tempo de fluoroscopia**	Tempo de fluoroscopia reduzido (DMP: -0,22; IC 95%: -0,32 a -0,12, p<0,0001).	Maior tempo de fluoroscopia (DM: 5,71 minutos; IC 95%: 1,13–10,30, p=0,01).
**IVPP**	Aumento da IVPP (OR: 0,47; IC 95%: 0,34–0,64, p<0,00001).	Não relatado explicitamente.
**RVP aguda**	RVP aguda reduzida (OR: 0,45; IC 95%: 0,35–0,58, p<0,00001).	Não relatado explicitamente.
**Segurança (Complicações)**	Lesão térmica esofágica reduzida (OR: 0,48; IC 95%: 0,30–0,77, p=0,002).	Nenhuma diferença significativa nas complicações periprocedimentais (RR: 1,20; IC 95%: 0,59–2,44).
**Principais descobertas**	A HPSD é mais eficiente e seguro que a LPLD, com menor recorrência de FA, tempos de procedimento mais curtos e menos complicações.	A PFA oferece tempos de procedimento mais curtos em comparação à ablação térmica, com segurança e eficácia comparáveis.
**Limitações**	ECRs limitados, heterogeneidade nos protocolos de ablação e potencial viés em estudos retrospectivos.	Populações heterogêneas, número limitado de estudos e potencial sobreposição nos dados dos pacientes.
**Tratamento de TAs focais e adicionais**	Rotina	Não é possível com o dispositivo atual
**Possibilidade de adicionar CNA**	Sim	Não
**Denervação Vagal**	Mais alta	Mais baixa
**Conclusão**	A ablação HPSD é superior à LPLD na redução da recorrência de FA e na melhoria da eficiência do procedimento.	A PFA é uma alternativa promissora à ablação térmica, com tempos de procedimento mais curtos e resultados comparáveis.

ATA: Taquiarritmias atriais; IVPP: isolamento das veias pulmonares de primeira passagem; RVP: Reconexão da veia pulmonar; PFA: Ablação de campo pulsado; HPSD: Alta potência de curta duração; LPLD: Baixa potência de longa duração; CNA: Cardioneuroablação; ECR: Ensaio clínico controlado randomizado; DMP: Diferença média padrão; DM: Diferença média; OR: Odd Ratio; RR: Razão de risco.

As duas metanálises se concentram em diferentes estratégias de ablação para FA: ablação por HPSD e PFA.^11 , 12^ Ambos os estudos têm como objetivo avaliar a eficácia, segurança e eficiência procedimental dessas técnicas de ablação em comparação aos métodos tradicionais.

## Conclusão

Ambos os métodos destacam os avanços nas técnicas de ablação de FA, com foco na melhoria da eficiência e segurança do procedimento. A metanálise HPSD demonstra que a ablação por radiofrequência de alta potência e curta duração reduz a recorrência de FA e os tempos de procedimento, ao mesmo tempo em que minimiza complicações como lesão esofágica. Por outro lado, a metanálise PFA mostra que a PFA oferece tempos de procedimento mais curtos em comparação à ablação térmica tradicional, sem diferença significativa em complicações ou recorrência de FA. Finalmente, uma questão altamente relevante que não foi adequadamente considerada nesses estudos é a extensão da denervação vagal resultante dessas técnicas. Está bem estabelecido que um maior grau de denervação vagal,^13^ incluindo a potencial incorporação de cardioneuroablação^14 , 15^ está fortemente associado a uma redução significativa nas taxas de recorrência a longo prazo.
