# Supplementary material for: Ablação de Curta Duração de Alta Potência ou Ablação de Campo Pulsado: Qual é a Melhor Escolha para Ablação da Fibrilação Atrial?
Source: Arq Bras Cardiol. 2025 Mar 25;122(2):e20250057. [Article in Portuguese] doi: 10.36660/abc.20250057 (PMC12129479; doi:10.36660/abc.20250057)
Supplement: Translation

# Translation: High-Power Short Duration or Pulsed Field Ablation: What is the Best Choice for AF Ablation?

**DOI:** 10.36660/abc.20250057i

**Authors:** José Carlos Pachon-M, Enrique I. Pachon-M, Tasso J. Lobo, Tomas G. Santillana-P, Carlos Thiene C. Pachon

**Affiliations:** 1 São Paulo SP Brazil Faculdade de Medicina da Universidade de São Paulo, São Paulo, SP – Brasil; 2 São Paulo SP Brazil Hospital do Coração, São Paulo, SP – Brasil

**Keywords:** Catheter Ablation, Cardiac Arrhythmias, Atrial Fibrillation, Irreversible Electroporation Therapy

We commend the authors for their valuable contribution to the study *"Atrial Fibrillation Catheter Ablation: Electroporation Against High-Power Short Duration Radiofrequency,"* ^1^which investigates the efficacy and safety of two distinct ablation techniques — high-power short-duration radiofrequency (HPSD)^2^and pulsed-field ablation (PFA)^3^— for the treatment of symptomatic atrial fibrillation (AF). Conducted at a single center in 2022, the study included 101 patients, 75% male, with a mean age of 61 years. This study is particularly timely and relevant, as both HPSD and PFA are relatively new AF ablation techniques that necessitate comprehensive evaluation to better establish their clinical utility, indications, and risk profiles in comparison with well-established conventional technologies.

Results indicated that both techniques effectively achieved pulmonary vein isolation (PVI), with HPSD showing significantly lower fluoroscopy time compared to PFA (5 vs. 13 minutes). However, HPSD had a longer procedure duration (97 vs. 88 minutes). Complications were minimal, with only one major complication (cardiac tamponade) occurring in the PFA group.

During follow-up averaging 384 days, 75% of patients maintained sinus rhythm, with AF recurrence observed in 25% of cases. Although PFA demonstrated shorter procedure times and potentially lower recurrence rates, both methods proved to be safe and effective for AF management. This study highlights the ongoing evaluation of new ablation technologies in clinical practice.

While HPSD is a modification of the well-known radiofrequency (RF) ablation,^4^PFA is an emerging non-thermal technique for AF ablation that utilizes high-voltage electrical pulses (up to 2000V) to induce irreversible electroporation, selectively targeting cardiac tissue while minimizing damage to surrounding structures.^5 , 6^Recent studies have primarily focused on the safety and efficacy of PFA, with particular attention to thromboembolic events.

In the PULSED AF pivotal trial,^6^PFA demonstrated a low rate of primary safety adverse events (0.7%), with no reported cases of PV stenosis, phrenic nerve injury, or esophageal injury. The study concluded that PFA provides effectiveness consistent with established ablation technologies while offering a favorable safety profile.

However, recent developments have raised concerns regarding thromboembolic risks associated with PFA.^3 , 7^Johnson & Johnson temporarily paused sales of its PFA system, Varipulse,^8 - 10^after four patients experienced neurovascular events, identified as strokes, following treatment. This incident has prompted increased scrutiny of PFA systems and highlighted the need for a thorough evaluation of their safety profiles.

These events underscore the importance of ongoing research and post-market surveillance to understand the thromboembolic risks associated with PFA fully. While early studies indicate a favorable safety profile, recent adverse events suggest that further investigation is necessary to ensure patient safety.

In summary, while PFA offers promising advantages in AF ablation, including reduced collateral damage and efficient lesion formation, recent reports of thromboembolic events highlight the need for continued vigilance and research to assess and mitigate associated risks fully.

As the current study compared HPSD vs. PFA,^1^it is highly appropriate to evaluate each of these technologies individually in relation to conventional RF catheter ablation. Table 1 provides an overview of the most recent meta-analyses assessing these technologies.

**Table 1:** Comparison of Meta-analysis of AF ablation using HPSD and PFA

Parameter	HPSD Meta-analysis (Xu et al., 2022)^11^	PFA Meta-analysis (Aldaas et al., 2024)^12^
**Objective**	Compare HPSD (>40 W) vs. LPLD ablation for AF, focusing on efficacy, safety, and procedural efficiency.	Compare PFA vs. thermal ablation (RF/cryo) for AF, focusing on procedural efficiency, safety, and efficacy.
**Number of Studies**	15 studies	6 studies
**Total Patients**	3,255 patients (1780 HPSD, 1475 thermal ablation/RF/cryo)	1,012 patients (441 PFA, 571 thermal ablation/RF/cryo)
**Primary Outcomes**	ATAs recurrence, AF recurrence, FPI, acute PVR, procedural time, fluoroscopy time, ETI, complications.	Procedural time, fluoroscopy time, periprocedural complications, AF recurrence.
**Recurrence of AF/ATAs**	Lower recurrence of ATAs at 1-year follow-up (OR: 0.49; 95% CI: 0.35–0.67, p<0.0001).	No significant difference in AF recurrence (RR: 0.64; 95% CI: 0.31–1.34).
**Procedural Time**	Shorter procedural time (SMD: -0.95; 95% CI: -1.06 to -0.85, p<0.00001).	Shorter procedural time (MD: -21.95 minutes; 95% CI: -33.77 to -10.14, p=0.0003).
**Fluoroscopy Time**	Reduced fluoroscopy time (SMD: -0.22; 95% CI: -0.32 to -0.12, p<0.0001).	Longer fluoroscopy time (MD: 5.71 minutes; 95% CI: 1.13–10.30, p=0.01).
**FPVI**	Increased FPVI (OR: 0.47; 95% CI: 0.34–0.64, p<0.00001).	Not explicitly reported.
**Acute PVR**	Reduced acute PVR (OR: 0.45; 95% CI: 0.35–0.58, p<0.00001).	Not explicitly reported.
**Safety (Complications)**	Reduced esophageal thermal injury (OR: 0.48; 95% CI: 0.30–0.77, p=0.002).	No significant difference in periprocedural complications (RR: 1.20; 95% CI: 0.59–2.44).
**Key Findings**	HPSD is more efficient and safer than LPLD, with reduced AF recurrence, shorter procedural times, and fewer complications.	PFA offers shorter procedural times compared to thermal ablation, with comparable safety and efficacy.
**Limitations**	Limited RCTs, heterogeneity in ablation protocols, and potential bias in retrospective studies.	Heterogeneous populations, limited number of studies, and potential overlap in patient data.
**Treating focal additional TAs**	Routine	Not possible with the current device
**Possibility of adding CNA**	Yes	No
**Vagal Denervation**	Higher	Lower
**Conclusion**	HPSD ablation is superior to LPLD in reducing AF recurrence and improving procedural efficiency.	PFA is a promising alternative to thermal ablation, with shorter procedural times and comparable outcomes.

ATA: Atrial Tachyarrhythmias; FPVI: First-Pass pulmonary vein isolation; PVR: Pulmonary Vein Reconnection; PFA: Pulsed Field Ablation; HPSD: High-Power Short-Duration; LPLD: Low-Power Long-Duration; CNA: Cardioneuroablation; RCT: Randomized Controlled Trial; SMD: Standard Mean Difference; MD: Mean Difference; OR: Odds Ratio; RR: Risk Ratio.

The two meta-analyses focus on different ablation strategies for AF: HPSD radiofrequency ablation and PFA.^11 , 12^Both studies aim to evaluate the efficacy, safety, and procedural efficiency of these ablation techniques compared to traditional methods.

## Conclusion

Both methods highlight the advancements in AF ablation techniques, focusing on improving procedural efficiency and safety. The HPSD meta-analysis demonstrates that high-power, short-duration radiofrequency ablation reduces AF recurrence and procedural times while minimizing complications like esophageal injury. On the other hand, the PFA meta-analysis shows that pulsed field ablation offers shorter procedural times compared to traditional thermal ablation, with no significant difference in complications or AF recurrence. Finally, a highly relevant issue that has not been adequately considered in these studies is the extent of vagal denervation resulting from these techniques. It is well established that a greater degree of vagal denervation,^13^including the potential incorporation of cardioneuroablation^14 , 15^is strongly associated with a significant reduction in long-term recurrence rates.
